# A Case of Pleuro-Pneumonia of the Right Lung; Bronchitis of the Left, Complicated with Inflammation of the Liver, Followed by Fatal Pericarditis

**Published:** 1849-03

**Authors:** David W. Yandell

**Affiliations:** Louisville, Ky.


Art. VIII.—*A Case of Pleuro-Pneumonia of the right Lung; Bronchitis of the left, complicated with Inflammation of the Liver, followed by fatal Pericarditis.—Autopsy.* By David W. Yandell, M.D., of Louisville, Ky.

Lavinia, a negress, belonging to Mrs. S., æt. 27 years, of strong constitution and robust habit, who, with the exception of one or two attacks of autumnal fever several years ago, and a slight inflammation of the surface of the liver in December last, had enjoyed good health, was seized on the 11th of January, after considerable exposure during the Christmas holidays while laboring under a severe catarrhal affection, with great pain and a sense of constriction in the chest, difficulty of breathing, and the other rational signs of pulmonary inflammation.

On Friday, the 12th, when I first saw the patient, her pulse was 160, hard and incompressible; skin dry and hot; intellectual faculties exceedingly obtuse; bowels constipated; urine high colored and scanty; tongue furred, red at edges and tip; thirst distressing; respiration 60 a minute, painful, laborious, and jerking; cough frequent, deep, and hard, attended by a metallic sort of noise, and followed by the copious expectoration of a viscid, tenacious, ropy mucus, some portions of which were perfectly transparent, others frothy, and others again of a sanguineous hue. She complained of the most distressing pain in the whole right side of the chest, extending to the hypochondrium, and most intense in this region and the lower lobe of the corresponding side, augmented by pressure, coughing, and respiration.

*Physical signs.*—Percussion revealed dullness extending from the mammæ to the base of the right lung. Resonance natural on the left side.

*Auscultation.*—Anteriorly, signs of consolidation were distinguished over the superior half of the dullness; absence of the respiratory murmur alone over the inferior half. Posteriorly, the same phenomena were present over nearly the same space, though I thought I could detect crepitation extending an inch, or an inch and a half lower down than on the anterior surface. Both anteriorly and posteriorly, however, the signs of engorgement mingled with sibilant rhonchus were perceptible from the superior margin of the dullness to the summit of the lung. The friction sounds were very evident from the right arm-pit to the superior margin of the liver, heard most distinctly from before backwards, and were most intense along the side of the spinal column.

The only thing noticeable on the left side of the chest was the intense puerility of the respiration.

I employed venesection to the amount of forty ounces, and abstracted ten ounces more by cups. The blood presented a highly inflammatory appearance, and even while flowing from the arm the dyspnea was considerably relieved. I ordered a flaxseed poultice to be placed over the inflamed lung, and prescribed the following:

℞. Hydrarg. chlorid. mitis,
Pulv. ipecac. comp. ää grs. x.
Ant. et potass. tartrat. grs. iv.
M. Divide in chart. viij.

One every hour till bedtime, when the patient was to take mist. camphor, aq. ammon. acet, ää ℥ss., vin. ant., tinct. opii. ää gtt. xx. As a demulcent, diuretic, and refrigerant, I allowed the patient to drink freely of a solution of bitart. potass. in slippery elm water with ice, and also permitted her to use ice *ad libitum*.

13th. The condition of the patient unaltered. She had passed a restless and painful night, coughing almost incessantly, and drinking every few minutes of the cream of tartar water. Her bowels had been moved twice; the discharges were profuse, dark colored and offensive. The kidneys had been well acted upon. The pulse was not less frequent or full; the skin still dry and hot; pain acute and distressing; cough frequent and painful; respiration 50 per minute; intellectual faculties obtuse; countenance dull and stupid. Physical signs the same.—Venesection and cups repeated; patient immediately after placed in a tepid bath, where she remained half an hour, and on coming out expressed herself as feeling greatly relieved of the pain in her side and the difficulty of breathing. I ordered cloths wrung out of tepid water to be constantly applied to the chest, and prescribed the following:

℞. Hydrarg. chlorid. mit. grs. xxiv.
Pulv. scill. ʒss.
Ant. et potass. tartrat. grs. vj.
Pulv. ipecac. comp. grs. xx.

Ft. pil. xviij. One to be taken every hour. The cream of tartar water and ice to be continued.

14th. Patient slept a little last night; was called up several times to stool, but the discharges were mixed with so large a quantity of urine that their character could not be ascertained. She had been kept very sick by the pills, and had vomited once or twice; respiration 40, less painful and jerking; pulse 142, less full and hard; skin dry and hot; tongue furred in center, red at edges and tip; cough more frequent and a little less painful; tenderness over the chest somewhat diminished; not so in the hypochondrium, where it had increased in intensity and now extended over the stomach into the left hypochondrium.

The physical signs, save the friction sounds which were no longer heard, remained the same. A yellowness of the conjunctiva now became very apparent. I ordered the calomel, squills, tart. antimony, and Dover's powder to be continued in the proportion of the day before, changing only the form, giving them now in powder instead of pill, with the addition of ʒiv of nitrat. potass. The bath to be repeated; the warm cloths to be constantly applied; sinapisms to be used over the stomach; continue tartar water and ice.

15th. Patient passed a very uncomfortable night, and when I saw her at 9 o'clock, A.M., she complained of suffering almost beyond endurance from the pain in the region of her liver and stomach. Her bowels had not been moved; her kidneys continued secreting very actively; and in every other respect her condition was the same as on the day before. The tartar emetic and calomel powders to be continued, increasing the quantity of the former to eight grains. To relieve the pain and assist diaphoresis I ordered a pill of the following:

℞. Ext. hyoscyam. grs. x.
Pulv. ipecac. grs. v.
Ant. sulphur. præcip. grs. vj.
Syrup q. s.

ut. ft. pil. x. One every hour, watching their effect. The warm cloths and sinapisms to be continued.

The patient having become tired of the tartar water, I prescribed

℞. Succ. limon. recent. ℥iss.

Potass. carbonat., q. s., ad saturand.

Sacc. albi ʒj.

Spts. æther. nitric, ʒj.

Aq. menth. ℥iij. M.

A tablespoonful every two hours.

16th. Patient passed a restless night; bowels moved four or five times; dark colored stools; very large quantities of urine; skin dry and burning; thirst very distressing; pulse 130; pain and tenderness over stomach and liver undiminished; over the chest very much so, indeed almost entirely gone, except a deep-seated pain just below the right nipple. Respiration 45, less laborious, less painful, fuller, and performed more by the thorax than previously; sputa unaltered; cough more paroxysmal in its character, less constant, brought on by turning over or rising up in bed. Continued prescription of day before.

17th. Had passed a restless night; four or five dark colored stools; large secretion of urine; pulse 132; skin less hot and dry than day before; less stupid, and more irritable. Physical signs on right side unchanged; on left, the puerile respiration has given place to the sonorous and sibilant rhonchi, mingled with an occasional mucous rhonchus. The pain less over the chest, liver and stomach; still considerable tenderness over two last. Respiration 40, but less laborious.

Without relating further in detail the history of the patient, I will remark that her condition continued materially the same, as was also the treatment, except that the tartar emetic was increased to twelve grains in the twenty-four hours, and that ten grains of quinine were administered in the morning and evening, until Sunday, the 21st, when the febrile reaction considerably abated, the constitutional effects of the mercury became apparent, the bronchitis began to disappear, the signs of consolidation commenced slowly receding, and the inflammation of the liver showed a disposition to subside.

I now resorted to large and repeated blisters over the whole of the right side, diminished the amount of mercury, used larger quantities of the pulv. ipecac. comp., and controlled the activity of the circulation by means of the tartarized antimony and quinine. In the course of the same week, I had the satisfaction of seeing the pneumonia almost entirely resolved except over a limited surface immediately next the spinal column, where it resisted constant counter-irritation and well directed constitutional means for five days longer, when it also disappeared, and I pronounced the patient convalescent. This was on Friday, the 25th, two weeks from the time I first saw the patient. The yellowness of the conjunctiva became less and less vivid until it, along with the principal phenomena of inflammation of the liver, almost entirely subsided, though there still remained a slight degree of tenderness on pressure over the diseased organ.

For four days the patient was comparatively free from pain, eat with some relish small quantities of well boiled rice and mush and milk, and drank toast water. On Friday she had considerable hemorrhage from the nose, and on that night her menses appeared, being a week in advance of the usual period. On Saturday afternoon I found her laboring under even more violent febrile reaction than at any previous time. Her pulse was 160, small, weak, and jerking; her countenance exceedingly anxious; she complained of a great sense of uneasiness in the precordial region, where the most distressing pain is excited by pressure, change of posture, cough, and deep inspiration, radiating to the left shoulder and down the same arm to the fingers' points.

Not to continue longer upon the rational symptoms, and to dismiss the physical signs as summarily, auscultation revealed the exocardial bellows sound, clear, loud, and prolonged; the beats of the heart tumultuous and unequal. I practiced a small bleeding, applied cups over the diseased organ, followed by blisters that were sprinkled with powdered digitalis; used calomel, opium, digitalis, quinine, tartar emetic, and, for a few days, thought the patient was improving; but on Monday she grew very rapidly worse, became delirious, and, after both her inferior and superior extremities had been cold for twelve hours, expired on Tuesday morning.

*Autopsy, twenty-four hours after death. Chest.*—The right pleura was attached by firm, recent, and very thick adhesions to the diaphragm, and to the ribs from the seventh to the third, inclusive. The pulmonary tissue of the same side appeared throughout firmer, heavier, more elastic, and of a redder hue than natural—all parts of it were entirely permeable to the air. About the center of the middle lobe there was a patch of the size of a dollar, or larger, which still retained the characters of hepatization; immediately around this the reddish shade of which I have made mention, was more evident than elsewhere. Left lung in every respect healthy. The vessels on the external surface of the heart were turgid with blood. On opening the sac of the pericardium it was found to contain about an ounce or an ounce and a half of yellowish looking serum. Towards the point of the heart it was partially but feebly adherent to this organ. Over the whole extent of its surface it was covered to the depth of from one to two lines and a half, with firm coagulable lymph, which manifested in several different places, besides the point of the heart, a strong disposition to become organized; indeed, there were no less than four or five small bands stretched across from one point to another, drawing the heart and its investment closely in contact, and retaining them in that position with considerable force. The whole of the internal structure of the heart, valves, membrane, etc., were in every respect healthy. The substance of the organ appeared somewhat though not palpably softened. The liver was considerably enlarged, engorged throughout, of a deep jaundiced hue, and its external surface, for about two lines into the substance of the organ, was hardened, offering some resistance to the knife. The left lobe was greatly indurated, grating under the knife. The other organs of the body were healthy.

*Remarks.*—During the progress of this case, I had an opportunity of exhibiting it to a number of medical students belonging to my class. The subject of my lectures was one which the case was well fitted to illustrate, and the following remarks, made before my class on its diagnosis, will not, perhaps, be deemed inappropriate in the present connection. I demonstrated to them the lesions of the heart, to which the fatal result was mainly attributable.

You will remember, I remarked, that, when speaking of pneumonia of the second degree, or hepatization, I told you it was often a very difficult matter to determine between it and inflammation of the liver accompanied by enlargement. The history of the case is very often of great service to us, and was so in the present instance. The girl had suffered from an attack of inflammation of the surface of the liver about the middle of December, and though she had been able to resume her work as house-servant, she continued to complain of a sense of uneasiness in her right side, and lost her appetite and spirits. From the color of the skin of the African, the jaundiced hue of the surface, which is observable in the white when laboring under disease of the liver, cannot be distinguished; but, fortunately, the color of the conjunctiva in health and its discoloration when the escape of bile through the proper channels is impeded, are the same in both races.

Pain in the right shoulder, though by no means always present in inflammation of the liver, is not the less valuable when it does exist. Pain and tenderness over the diseased organ, augmented by pressure, change of posture, coughing, and deep breathing; stupor, somnolency, and decubitus on the diseased side, are some of the more prominent of the symptoms of hepatitis. The symptom which is next in importance to the pain and tenderness is the tumified condition of the organ. A practical hint that may be of service to you is, that whenever you wish to examine the liver or spleen it is very important to have the intestines empty and the belly flaccid:

for when they are distended by either air or fecal matter, it is next to impossible to obtain a correct idea of the volume of these organs. When the bowels are empty and the belly flaccid, you will, in most cases, be able to detect hepatic enlargement by the fullness in the right hypochondriac and epigastric regions, and the edge of the liver protruding more or less below the costal cartilages. In some instances,. the ribs appear bulged out; but (and I wish you to bear this in mind, for it will be of great assistance to you in the diagnosis between hepatic and pulmonary disease) the intercostal spaces preserve their relative positions with respect to them. In hydrothorax, on the other hand, the intercostal spaces are obliterated, and if the effusion be very great they are even bulged out.

You ask me, by what means I was able to determine, in the case of Lavinia, where the dullness, dependent on the liver, ended superiorly, and that produced by hepatization commenced? Or, in other words, how was I enabled to tell how much of the thoracic dullness was produced by hepatitis, and how much by pneumonitis? By percussion alone I could not tell; it was inadequate. But by applying my ear to the chest while the patient was lying down, and listening attentively for the inferior limits of the signs of pneumonia, and then carefully bearing them in mind, or tracing a line upon the chest at this point, the patient was desired to sit up and take a deep inspiration, when the pneumonic disease was found to reach quite a perceptible distance farther down than before; in other words, by the expansion of the lung by a full inspiration, the liver was in some degree displaced, driven down; and where, before, I heard no sound at all, I now discovered a very loud blowing noise, which is one of the signs of consolidation of the lung.

The physical signs of pneumonia vary according to the period of the disease. The first period is, according to Stokes, characterized by rudeness of the respiration; the second, or that of engorgement, by the crepitant rhonchus; the third, or that of hepatization, by the bronchial respiration and bronchophony; dullness on percussion is also observed at this period; the fourth, or that of softening, by the mucous rhonchus. The inflammation in the present instance attained only the third degree. The progress of the disease was this; first, there was dryness of the lining membrane of the air cells of the lungs, which was revealed by the rudeness of the respiration. The inflammation increased; engorgement of the cells ensued, and crepitation was the consequence. The inflammation continued to advance, solidification was effected, and the cells, closed up, were no longer permeable to the air, which, being confined to the bronchial tubes, made its way through them with a loud rushing noise that has been aptly styled the tubal respiration. The corresponding alteration on the part of the voice is called, with equal expressiveness, bronchophony.

As the inflammation subsided, the signs of solidification gave place to those of engorgement; these, in their turn, yielded to that of simple congestion; and this grew less and less distinct until finally the normal vesicular murmur alone could be heard. These *recurrent* phenomena only obtain when the inflammation has stopped short of the fourth stage, or that of softening.

Not to detain you longer, I will conclude by adding that the pleurisy was revealed to us by the friction sound, of which there are two varieties—the soft or *grazing*, and hard or *scraping*, friction sounds. And, finally, the inflammation of the bronchi was evinced by the sonorous, sibilant, and mucous rhonchi.

Feb. 21, 1849.

